# A comparative study on L-thyroxine treatment and sesame oil supplementation in experimentally induced hypothyroidism in rats

**DOI:** 10.3389/fphys.2025.1606528

**Published:** 2025-11-24

**Authors:** Noha N. Lasheen, Sara Shawky, Noha Gaber, Abd El-Hamid A. Mohamed

**Affiliations:** 1 Department of Medical Physiology, Faculty of Medicine, Ain Shams University, Cairo, Egypt; 2 Department of Basic Medical Sciences, Faculty of Medicine, Galala University, Suez, Egypt; 3 Department of Anatomy and Embryology, Faculty of Medicine, Ain Shams University, Cairo, Egypt

**Keywords:** cardiac fibrosis, hepatic fibrosis, hypothyroidism, L-thyroxine, sesame oil, oxidative stress

## Abstract

**Background and Aims:**

Natural antioxidants have gained increasing attention in medical and nutritional research. Sesame oil, a supplement widely recognized for its anti-inflammatory and antioxidant properties, remains underexplored with respect to its potential role in hypothyroidism management. Levothyroxine is currently the mainstay of therapy for hypothyroidism. Based on this, we aimed to demonstrate the effects of different treatments on the systemic parameters, including the liver and heart, of hypothyroid rats and to elucidate the underlying mechanisms.

**Methodology:**

Adult female Wistar rats (n = 66) were randomly allocated into control, sesame oil-treated euthyroid, propylthiouracil-induced hypothyroid, L-thyroxine-treated hypothyroid, sesame oil-treated hypothyroid, and combined treated hypothyroid groups. After 8 weeks, arterial blood pressure values were measured using a noninvasive rat tail sphygmomanometer. On the day of sacrifice and after overnight fasting, rats were anesthetized with pentobarbitone, and electrocardiograms were recorded. Separated plasma samples were used to measure the thyroid hormone levels, cardiac and liver enzymes, lipid profile, oxidative stress markers, and interleukin-6. Hepatic low-density lipoprotein receptor concentration and hepatic stearoyl-CoA desaturase 1 gene expression were determined, in addition to histopathological studies of heart and liver tissues.

**Results:**

Primary hypothyroidism was evident in the hypothyroid group, whereas all treated groups were euthyroid. Compared to the control group, the hypothyroid group exhibited systolic hypotension, diastolic hypertension, arrhythmia, higher cardiac enzymes, dyslipidemia, impaired liver functions, upregulated hepatic stearoyl-CoA desaturase 1 expression, lower hepatic low-density lipoprotein receptor concentration, cardiomyopathy, and focal hepatic fibrosis. Both L-thyroxine and sesame oil showed cardioprotective and hepatoprotective effects, whereas sesame oil exhibited greater lipolytic effects by enhancing low-density lipoprotein receptor concentration; both caused downregulated hepatic stearoyl-CoA desaturase 1 gene expression. Hypothyroid-induced oxidative stress was limited in all treated groups, whereas sesame oil had additional anti-inflammatory effects. Synergistic lipolytic effects and better control of diastolic blood pressure were observed in the hypothyroid group treated with the combination.

**Conclusion:**

Sesame oil has the potential to be utilized as an adjuvant therapy with L-thyroxine to counteract the cardiac and hepatic alterations induced by hypothyroidism. This is supported by its antioxidant, anti-inflammatory, and antisteatotic characteristics.

## 1 Introduction

Hypothyroidism, a common thyroid disorder, is diagnosed when the thyroid hormone levels are lowered, accompanied by high thyroid-stimulating hormone (TSH) levels (Udovcic et al., 2017). Hypothyroidism is more common in women than in men (Murgod and Soans, 2012). It may result from reduced thyroid hormone formation or as a consequence of improper treatment of hyperthyroidism (Milosevic et al., 2004), which is manifested as either subclinical or overt. Subclinical hypothyroidism occurs when the TSH concentration is raised above the upper limit of the reference range despite a normal free thyroxine (T4) level in the serum (Pasupathi and Latha, 2008).

Hypothyroidism alters the functions of various body organs (Prasad and Rao, 2012), causing cardiovascular disease, metabolic syndrome, and metabolic-associated liver disease (MALD) (Taylor et al., 2013). Moreover, overt hypothyroidism adversely affects cardiovascular morbidity and mortality (Klein and Ojamaa, 2001).

The relationship between thyroid hormones and oxidative stress remains debatable. In an earlier study, free radical production was lowered in hypothyroidism due to reduced metabolism induced by the decline of thyroid hormone levels (Venditti et al., 1997). In hypothyroid states, oxygen demand is reduced, thereby protecting against reactive oxygen species (ROS)-induced tissue injury (Ísman et al., 2003). However, later studies reported that hypothyroidism might cause higher ROS generation and lower antioxidant capacity (Sarandol et al., 2005).

Long-term thyroid hormone treatment may result in pathological systemic effects (Gullo et al., 2011). Thus, there is a need to incorporate plant-based therapy, a “back to nature” approach, instead of relying solely on synthetic drugs, to reduce the adverse effects that may at times be more harmful than the disease itself (Khalawi et al., 2013).

Sesame oil has been used as a solvent for many hormones in experimental studies; however, it has many therapeutic effects (Hsu and Parthasarathy, 2017). Sesamin and sesaminol, the major phenolic constituents of sesame oil, demonstrated antioxidant, antihypertensive, anti-inflammatory, and antithrombotic effects (Sankar et al., 2005). Nonetheless, the molecular protective effects remain incompletely understood (Soliman et al., 2015).

Previous studies have not investigated the potential effects of sesame oil supplementation in hypothyroid status. Therefore, it is of interest to study the potential effects of sesame oil treatment in hypothyroidism alone or in combination with levothyroxine (L-thyroxine).

## 2 Aim of the work

This study demonstrated the systemic effects of experimental hypothyroidism on the cardiac and hepatic physiological, biochemical, and structural changes in Wistar rats and compared the impacts of treating hypothyroid rats with L-thyroxine alone to that with sesame oil, whether alone or combined with hormone replacement, as an adjuvant therapy.

## 3 Materials and methods

### 3.1 Animals

Sixty-six adult female Wistar rats, aged 12 weeks and initially weighing 140 g–180 g, were purchased from Animal Farm (Helwan), Egypt. The rats were housed in the Medical Ain Shams Research Institute (MASRI) (5 rats/cage) with suitable ventilation, 22 °C–25 °C temperature, and a normal light–dark cycle with free access to food (standard rat chow) and water *ad libitum*. All rats received appropriate human care based on the guidelines outlined in the “Guide for the Care and Use of Laboratory Animals.” This care was provided following the animal-use guidelines of the Ethical Committee of Ain Shams University, FMASU R116/2024, and the “National Institutes of Health guide for the Care and Use of Laboratory Animals” (NIH Publications No. 8023, updated in 2011, eighth edition).

Only female rats were used in this study because it was previously demonstrated that systemic effects of hypothyroidism are more prominent in females than in males (Karkoutly et al., 2020).

After 1 week of acclimation, rats were randomly and equally divided into six groups:

1. Control group (n = 11): this group received an intraperitoneal (i.p.) saline injection equivalent in volume to the injected propylthiouracil (PTU) in other studied groups. They were also given distilled water daily via gavage at a volume equivalent to the sesame oil administered to the other groups. This group served as the negative control.
2. Sesame oil-supplemented euthyroid group (n = 11): this group received an i.p. saline injection equivalent in volume to the injected PTU in other studied groups. They additionally received sesame oil (5 mL/kg B.W./day) by gavage for the last 4 weeks. They served as the positive control.
3. Hypothyroid group (initially there were 13 rats; two rats died during the experimental period, n = 11): this group was administered an i.p. injection of PTU (Sigma-Aldrich), dissolved in saline (10 mg/kg B.W/day), for 4 successive weeks (Baltaci et al., 2013). PTU injection was continued till the end of the experimental period, which was 8 weeks.
4. L-thyroxine-treated hypothyroid group (initially, there were 12 rats; one rat died during the experimental period, n = 11): they were rendered hypothyroid by the same protocol as the hypothyroid group. Then, thyroxine (T4) (Sigma-Aldrich) dissolved in saline was intraperitoneally injected (2 μg/100 g B.W/day) for another 4 weeks (Yuan and Yang, 1999), with continued PTU injection.
5. Sesame oil-treated hypothyroid group (initially there were 12 rats; one rat died during the experimental period, n = 11): they were also rendered hypothyroid similar to the hypothyroid group; thereafter, they received sesame oil by gavage (5 mL/kg B.W/day) for another 4 weeks (Saleem et al., 2014), with continued PTU injection.
6. Combined L-thyroxine and sesame oil-treated hypothyroid group (n = 11): they were rendered hypothyroid similar to the hypothyroid group, and then, they were treated by an i.p. injection of T4 and sesame oil by gavage for another 4 weeks, with continued PTU injection.

The experimental period lasted 8 weeks. During the first 4 weeks, hypothyroidism was induced in the untreated and three treated hypothyroid groups through PTU administration. In the treated hypothyroid groups, each respective treatment was administered for the following 4 weeks. The negative control group and the sesame oil-supplemented group received saline throughout the entire experimental period; however, the sesame oil-supplemented group also received sesame oil during the final 4 weeks of the experiment.

### 3.2 Drugs and chemicals

#### 3.2.1 Propylthiouracil (PTU)

Propylthiouracil (Sigma-Aldrich) was supplied as a powder and maintained at room temperature, 26 °C–27 °C.

#### 3.2.2 Sesame oil contents and extraction

Sesame oil, purchased from Sigma-Aldrich, was reported to contain beneficial fatty acids such as oleic and linoleic acids (Peng et al., 2015), as well as phytochemicals including tocopherol, phytosterol, lignan, and polyphenol with recognized antioxidant properties (Dar et al., 2015). The lignans, one of the major constituents of sesame oil, having a chemically methylenedioxyphenyl group, are sesamin, episesamin, sesaminol, and sesamolin (Gokbulut, 2010).

Laboratory methods for sesame oil extraction have been reported to use either supercritical CO_2_ (Doker et al., 2010) or a Soxhlet extractor with n-hexane solvent (Mohammed and Hamza, 2008).

### 3.3 Experimental procedures

At the end of the experimental period (8 weeks), all rats were subjected to arterial blood pressure measurement using a noninvasive small animal tail blood pressure system (NIBP200A, Biopac Systems Inc., United States).

On the day of sacrifice, overnight fasted rats were weighed and anaesthetized with an i.p. injection of pentobarbitone (40 mg/kg B.W.). When the stage of surgical anesthesia was reached, an ECG recording was performed using the ECG recorder Cardimax FX-2111 (Fukuda Denshi Co., Ltd., Japan). The heart rate, the R voltage, and the Q–T interval duration were calculated from lead II of the ECG tracing. The corrected Q–T interval (QT-c) was calculated according to the study by Goldschlager and Goldman (1984):

$$\text{QT}-c\text{ interval}=Q-T\;\text{interval}/\sqrt{(R}-R\;\text{interval}).$$

Following a midline abdominal incision, arterial blood samples were drawn from the abdominal aorta into heparinized tubes. The separated plasma samples were used for the subsequent determination of plasma levels of free triiodothyronine (T3), free T4, TSH, troponin I, and interleukin 6 (IL-6) using commercially available ELISA kits. In addition, creatine kinase (CK-MB), malondialdehyde (MDA), total antioxidant capacity (TAC), lipid profile, and plasma AST and ALT activities were determined using commercially available colorimetric kits. All assays were performed according to the manufacturer’s instructions.

The median liver lobe specimens were preserved at −80 °C for the subsequent determination of LDL receptors in hepatic tissue using ELISA kits provided by Cell Biolabs, Inc., United States. Correspondingly, stearoyl-CoA desaturase 1 (SCD1) gene expression was assayed in hepatic tissues by real-time PCR. Samples of the heart and the left liver lobe were used for histopathological studies.

#### 3.3.1 RNA preparation and real-time qPCR analysis

Total mRNA was extracted from liver tissues using RNeasy kits with spin-column DNase digestion (Qiagen). Purity and concentration were determined with a Nanodrop 1000 spectrophotometer (Thermo Fisher Scientific). One μg of RNA was used to synthesize cDNA with a QuantiTect Reverse Transcription Kit (Qiagen) and diluted to 10 ng/μL. The expression of mRNA was determined using the RT^2^ qPCR Primer Assay for rat stearoyl-Coenzyme A desaturase 1 (SCD1), catalog no: 330001 (Qiagen), and SYBR green RT^2^ SYBR Green ROX™ qPCR Mastermix on an Applied Biosystems StepOnePlus RT-PCR system. PCR was mixed for one reaction. Component volume RT^2^ SYBR Green Mastermix was prepared as follows: 12.5 μL cDNA synthesis reaction, 1 μL RT^2^ qPCR primer assay (10 μM stock), 1 μL RNase-free water, and 10.5 μL total volume 25 μL. The PCR cycling conditions were adjusted according to the manufacturer’s instructions. The cycling program included an initial activation step for 10 min at 95 °C to activate HotStarTaq DNA Polymerase. The cycling process included the following: denaturation for 15 s at 94 °C, annealing for 30 s at 60 °C, and extension for 30 s at 70 °C for X40 cycles. PCR products were quantified fluorometrically using SYBR Green and were normalized to the housekeeping gene rat glyceraldehyde-3-phosphate dehydrogenase (GAPDH). Then, the calculation was performed relative to the control according to the following formula:

Target  amount=2−ΔΔCt.

Here, ΔΔCt = {[Ct (target gene) – Ct (GAPDH)] - [Ct (control) - Ct (GAPDH control)}.

Fold difference for gene expression was calculated as 2−ΔΔCT using the endogenous control genes (liver). The identity and purity of the amplified product were assessed by melting curve analysis at the end of amplification.

Histological examination of heart and liver tissues: the liver and heart were dissected out, and their tissue samples were fixed in neutral buffer formalin (NBF) for 24 h, processed using a graded ethanol series, and embedded in paraffin. Then, 5-μm-thick sections were obtained from the right lobe of the liver and the left ventricle and stained with hematoxylin and eosin (H&E) to study the histological structure and with Masson’s trichrome to detect collagen fibers (Bancroft and Gamble, 2008).

Immunohistochemical study: sections from the left ventricle myocardium and right lobe of the liver were additionally subjected to immunohistochemical staining with CD117 in the heart and CD68 in the liver (Zhou et al., 2010). All sections were examined under a light microscope (Olympus CX23).

Morphometric study and statistical analysis: in the studied groups, ten nonoverlapping fields were assessed using ImageJ software version 1.50i for the following parameters:

1. Collagen fiber area percentage (%), which was measured in Masson’s trichrome-stained liver and heart sections at a magnification of 400.
2. The number of CD68-positive Kupffer cells, which was counted in the liver sections of immunohistochemistry.
3. The number of CD117-positive progenitor cells, which was counted in the heart sections of immunohistochemistry at a magnification of 400.

### 3.4 Statistical analysis

All results in this study were expressed as the mean ± SEM. Statistical package for the social sciences (SPSS, Inc., Chicago, IL, United States) program, version 20.0, was used to compare the significance between each pair of groups, using one-way ANOVA and *post hoc* test. Differences were considered significant when p ≤ 0.05. In addition, two-way ANOVA for differences between the means of different groups was used to demonstrate the effects of hypothyroidism and sesame oil supplementation.

## 4 Results

Sesame oil-supplemented euthyroid rats had nonsignificant changes in all studied parameters compared to the control group, so they served as positive controls.

### 4.1 Thyroid function

**FIGURE 1:**
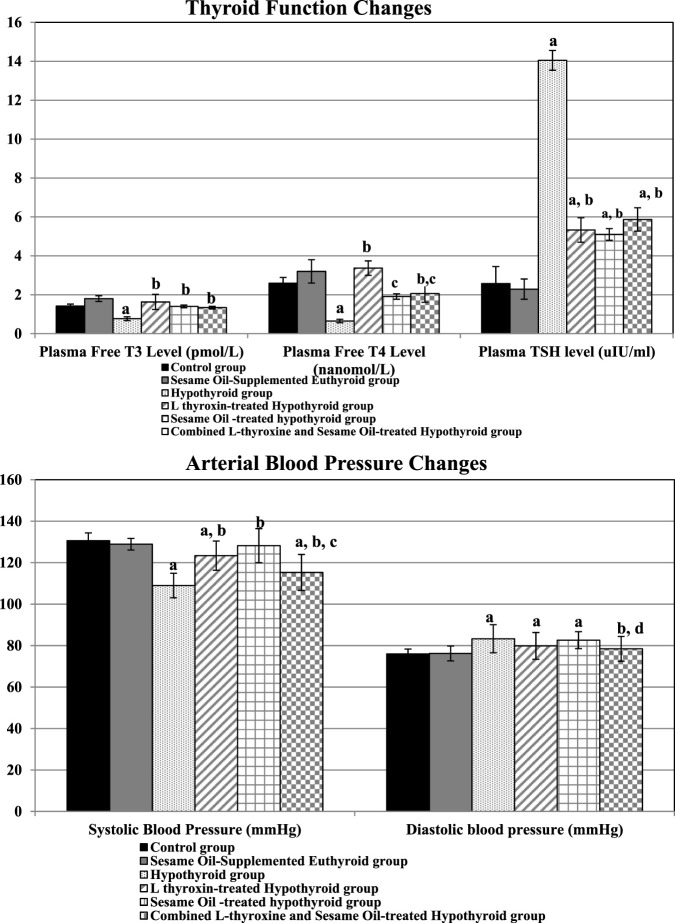
Changes in thyroid function and arterial blood pressure values in the different studied groups: a: significance from the control group by LSD at p ≤ 0.05. b: Significance from the hypothyroid group by LSD at p ≤ 0.05. c: Significance from the L-thyroxine-treated hypothyroid group by LSD at p ≤ 0.05. d: Significance from the sesame oil-treated hypothyroid group by LSD at p ≤ 0.05.

The PTU-induced hypothyroid group showed significantly lowered plasma T3 and T4 levels accompanied by significantly higher plasma TSH levels than the control rats. Meanwhile, all the treated hypothyroid groups (L-thyroxine-treated, sesame oil-treated, and the combined treated) were euthyroid compared to the controls despite the persistence of higher TSH levels. The plasma T4 level was significantly higher in the L-thyroxine-treated hypothyroid group than in either the controls or the hypothyroid rats, as shown in Figure 1.

### 4.2 Cardiovascular functions

#### 4.2.1 Blood pressure changes

The hypothyroid group exhibited significantly lowered systolic blood pressure and significantly higher diastolic blood pressure values than the controls.

All the treated hypothyroid groups exhibited significantly higher systolic blood pressure values than the hypothyroid rats. Compared to control rats, L-thyroxine treatment, either alone or in combination with sesame oil, significantly reduced systolic blood pressure.

On the other hand, diastolic blood pressure was still higher in the L-thyroxine- and the sesame oil-treated hypothyroid groups than in the controls. The diastolic blood pressure was significantly lowered only in the combined treated hypothyroid group compared to the hypothyroid rats, as shown in Figure 1.

#### 4.2.2 ECG changes

**FIGURE 2:**
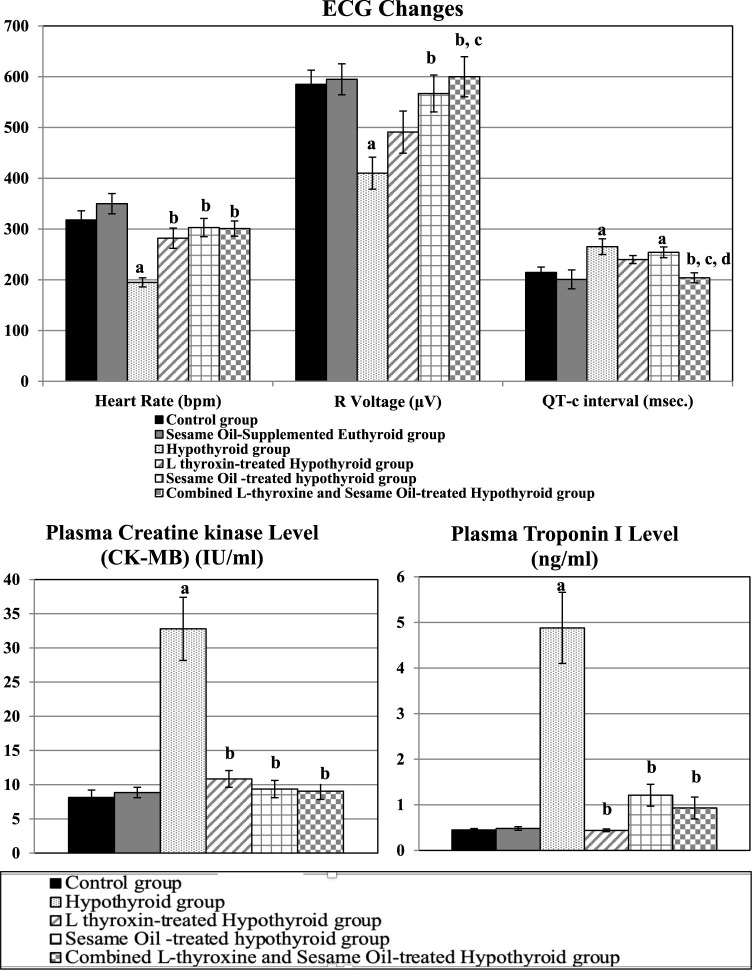
ECG changes and cardiac enzymes changes in the different studied groups: a: significance from the control group by LSD at p ≤ 0.05. b: Significance from the hypothyroid group by LSD at p ≤ 0.05. c: Significance from the L-thyroxine-treated hypothyroid group by LSD at p ≤ 0.05. d: Significance from the sesame oil-treated hypothyroid group by LSD at p ≤ 0.05.

The hypothyroid group exhibited significant bradycardia, significantly lowered R voltage, and significantly prolonged QT-c interval than the control rats. The heart rate was significantly elevated in all the treated hypothyroid groups than in the hypothyroid rats. Meanwhile, R voltage was significantly higher in the sesame oil-treated and the combined treated hypothyroid groups than in the hypothyroid rats. The QT-c interval was significantly shortened only in the combined treated hypothyroid group compared to the untreated hypothyroid ones. Significantly higher R voltage and significantly shortened QT-c interval were present in the combined treated hypothyroid group than in the L-thyroxine-treated hypothyroid group, as shown in Figure 2.

#### 4.2.3 Cardiac enzymes

Plasma levels of creatine kinase (CK-MB) and troponin I were significantly elevated in the hypothyroid group than in the control rats; however, both of them were significantly reduced in all the treated hypothyroid groups compared to the hypothyroid rats, as shown in Figure 2.

### 4.3 Liver functions

#### 4.3.1 Liver enzymes

**FIGURE 3:**
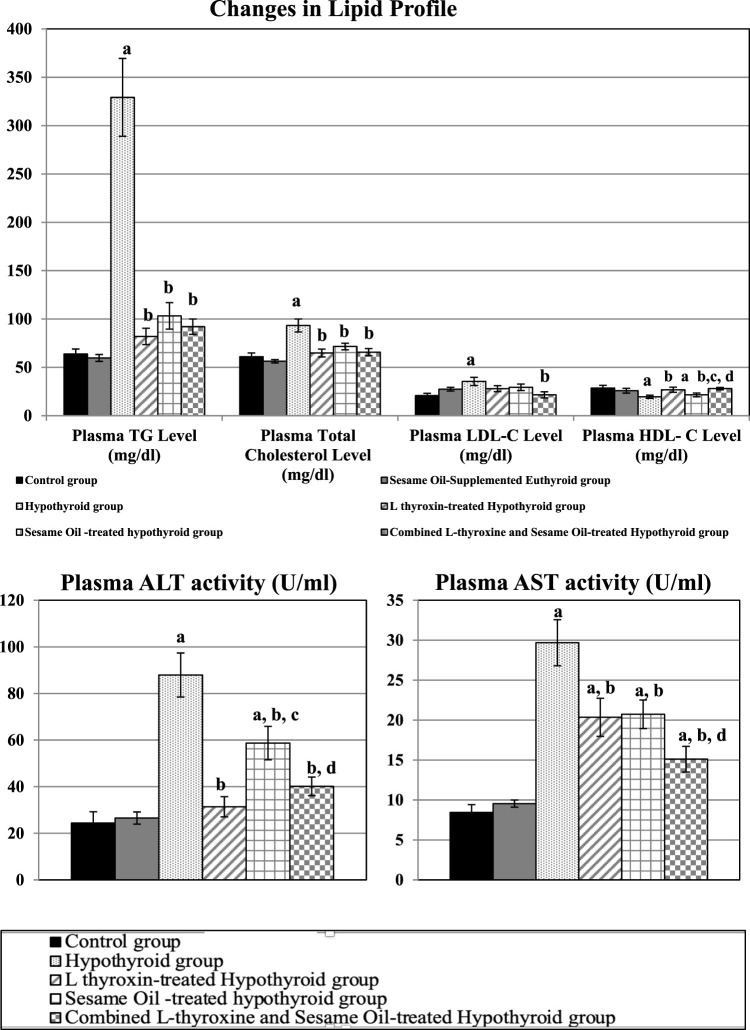
Changes in plasma lipid profile and liver functions in the different studied groups: a: significance from the control group by LSD at p ≤ 0.05. b: Significance from the hypothyroid group by LSD at p ≤ 0.05. c: Significance from the L-thyroxine-treated hypothyroid group by LSD at p ≤ 0.05. d: Significance from the sesame oil-treated hypothyroid group by LSD at p ≤ 0.05.

As demonstrated in Figure 3, plasma ALT activity was significantly higher in both the untreated hypothyroid and sesame oil-treated hypothyroid groups than in the control group. However, it was significantly lowered in all the treated hypothyroid groups compared to the untreated hypothyroid ones. On the other hand, plasma AST activity was significantly elevated in the untreated and all treated groups compared to the control rats; however, it was significantly reduced in all the treated hypothyroid groups compared to the hypothyroid rats.

#### 4.3.2 Lipid profile changes

As shown in Figure 3, the hypothyroid group showed significant dyslipidemia that manifested as significantly elevated plasma TG, total cholesterol, and LDL-cholesterol levels, accompanied by significantly lowered plasma HDL-cholesterol levels compared to the control ones. However, all the treated hypothyroid groups showed significant reductions in plasma TG and total cholesterol levels compared to the hypothyroid rats. Meanwhile, plasma LDL-cholesterol levels were significantly lowered only in the combined treated hypothyroid group compared to the hypothyroid rats. Plasma HDL-cholesterol was significantly higher in L-thyroxine and combined treated hypothyroid groups than in the hypothyroid rats, despite being significantly lower in the sesame oil-treated hypothyroid group than in the control ones.

#### 4.3.3 Hepatic LDL-receptor concentration

The concentration of hepatic LDL receptor was significantly reduced in both the untreated and L-thyroxine-treated hypothyroid groups as compared to the control groups. However, treatment with sesame oil alone or in combination with L-thyroxine caused a significant increase in the concentration of hepatic LDL receptor, as shown in Table 1.

**TABLE 1:** Changes in hepatic LDL-receptor concentration, hepatic SCD1 gene expression, and plasma IL-6 level in the different studied groups.

Groups	Control group	Sesame oil-supplemented euthyroid group	Hypothyroid group	L-thyroxin-treated hypothyroid group	Sesame oil-treated hypothyroid group	Combined L-thyroxine- and sesame oil-treated hypothyroid group
Hepatic LDL-R Conc. (pg/mL), (±SD)	75.2 ± 25.21	78.53 ± 24.26	24.48 ± 15.44	36.83 ± 14.37	79.8 ± 20.98	74.77 ± 26.54
a		NS	<0.001		NS	NS
b				<0.002	<0.001	<0.001
c				NS	<0.001	<0.002
Hepatic SCD1 Gene expression (fold)/ul, (±SD)	0.042 ± 0.03	0.038 ± 0.03	0.436 ± 0.028	0.08 ± 0.048	0.115 ± 0.11	0.025 ± 0.027
a		NS	<0.001	NS	NS	NS
b				<0.001	<0.001	<0.001
c					NS	NS
Plasma IL-6 Level (pg/mL) (±SEM)	122.88 ± 4.6	119.26 ± 3.45	152.39 ± 6.59	141.35 ± 5.38	135.04 ± 2.68	129.55 ± 3.62
a		NS	<0.001	<0.01	NS	NS
b				NS	<0.02	<0.002
c					NS	NS

Values are the mean ± SD/SEM of 11 rats in each group.

NS: nonsignificant.

^a^ Significance from the control group by LSD at p ≤ 0.05.

^b^ Significance from the hypothyroid group by LSD at p ≤ 0.05.

^c^ Significance from the L-thyroxine-treated hypothyroid group by LSD at p ≤ 0.05.

#### 4.3.4 Hepatic SCD1 gene expression

As demonstrated in Table 1, the hypothyroid group had significantly upregulated hepatic SCD1 gene expression compared to the controls, whereas it was significantly downregulated in all the treated hypothyroid groups compared to the hypothyroid rats.

### 4.4 Oxidative stress markers

**FIGURE 4:**
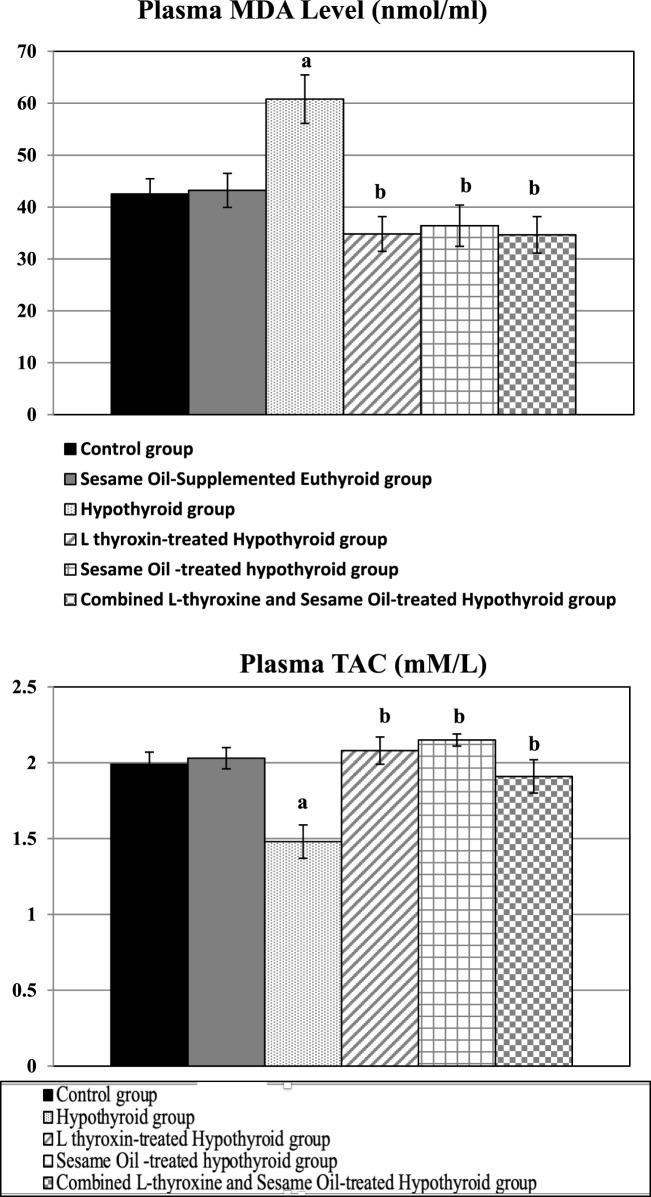
Changes of oxidative stress markers in the different studied groups: a: Significance from the control group by LSD at p ≤ 0.05. b: Significance from the hypothyroid group by LSD at p ≤ 0.05.

The hypothyroid group had prominent oxidative stress that manifested as significantly higher plasma MDA levels, accompanied by significantly lower plasma total antioxidant capacity (TAC), than the control rats. Nonetheless, these parameters were reversed in all the treated hypothyroid groups compared to the hypothyroid rats, as shown in Figure 4.

### 4.5 The inflammatory mediator IL-6 in the plasma

Inflammatory mediator IL-6 was significantly elevated in untreated and L-thyroxine-treated hypothyroid groups compared to the control ones. However, sesame oil treatment either alone or in combination with L-thyroxine caused a significant reduction in plasma IL-6 compared to the hypothyroid rats, as shown in Table 1.

**FIGURE 5:**
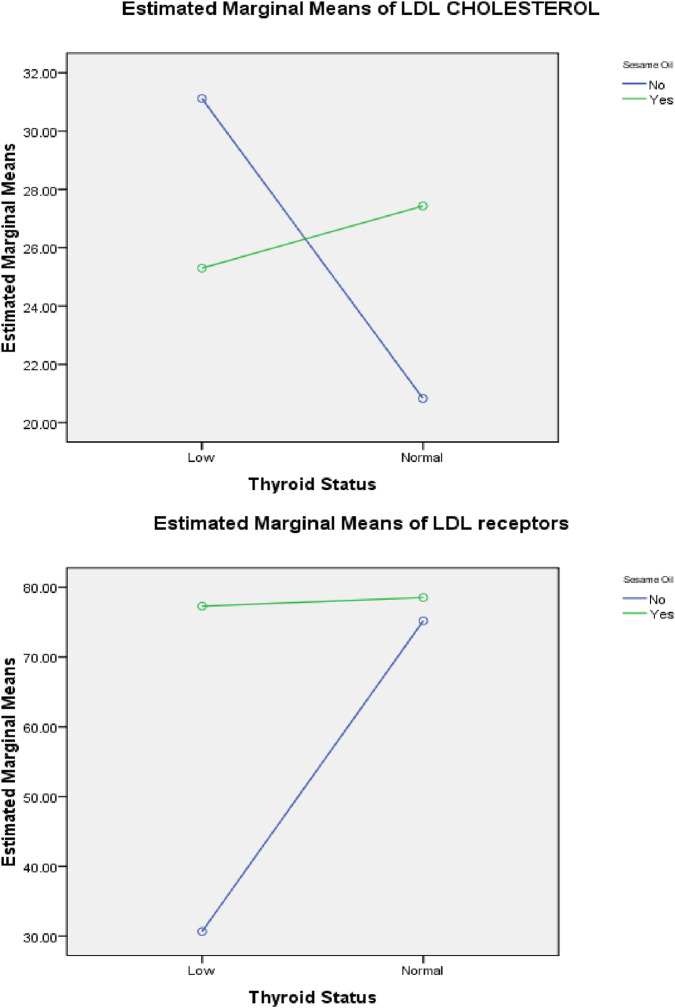
Effects of the interaction between thyroid status and sesame oil treatment on plasma LDL-cholesterol and hepatic LDL-R concentration.

**FIGURE 6:**
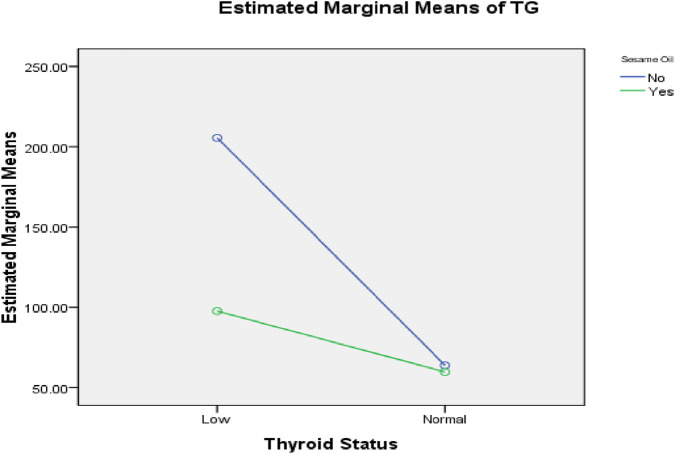
Effects of the interaction between thyroid status and sesame oil treatment on plasma TG and LDL cholesterol.

**FIGURE 7:**
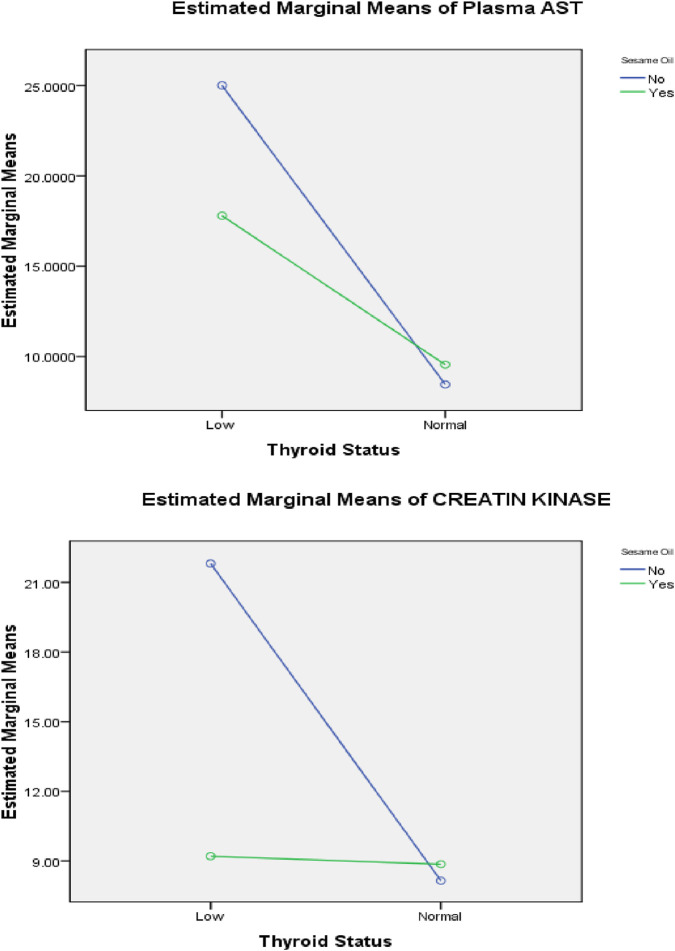
Effects of the interaction between thyroid status and sesame oil treatment on plasma AST activity and plasma creatine kinase (CK-MB) level.

**FIGURE 8:**
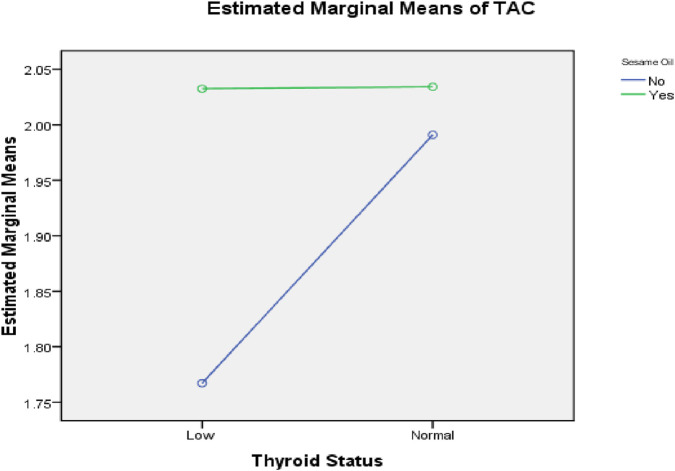
Effect of the interaction between thyroid status and sesame oil treatment on TAC.

Two-way ANOVA was performed to demonstrate the interaction between sesame oil and thyroid status, as shown in Figures 5–8. Without sesame oil, low thyroid status was associated with higher LDL cholesterol in the plasma and lower LDL receptors in the liver, whereas treated hypothyroid rats reaching normal thyroid status had lowered plasma LDL cholesterol and higher hepatic LDL receptors. With sesame oil, LDL cholesterol remains relatively stable regardless of the thyroid status, and hepatic LDL receptors stay high regardless of thyroid function.

Therefore, sesame oil appeared to normalize the effects of thyroid status on both plasma LDL cholesterol and hepatic LDL receptor measures, thus reducing the variations present in the group not receiving sesame oil.

In addition, rats without sesame oil supplementation exhibited significantly higher estimated triglyceride levels under low thyroid status (∼205 mg/dL) than those under normal thyroid status (∼65 mg/dL). Rats with sesame oil supplementation also had elevated plasma triglyceride levels in the hypothyroid status (∼98 mg/dL), but the increase was less notable than in rats without sesame oil supplementation. This indicates that hypothyroidism causes a marked increase in plasma triglyceride levels, and sesame oil appears to reduce this increase.

Furthermore, in hypothyroid rats without sesame oil supplementation, plasma HDL levels were lower at low thyroid status (∼23 pmol/L) and increased at normal thyroid status (∼28.5 pmol/L). Rats with sesame oil supplementation have higher HDL plasma levels at low thyroid status (∼25 pmol/L) than those without sesame oil supplementation and showed a mild increase at normal thyroid status (∼25.9 pmol/L). This suggests hypothyroidism lowers HDL levels, but sesame oil reveres this reduction.

Regarding liver functions, hypothyroid rats without sesame oil supplementation had significantly elevated AST levels compared to normal thyroid rats, whereas those with sesame oil treatment had significantly lower AST levels. This indicates that sesame oil reduces the elevation of AST caused by hypothyroidism.

Regarding cardiac biomarkers, hypothyroid rats without sesame oil supplementation showed much higher plasma creatine kinase levels than euthyroid rats. With sesame oil treatment, creatine kinase levels in hypothyroid rats were reduced and close to the levels present in euthyroid rats. This effect suggests that sesame oil markedly decreases the increased creatine kinase activity associated with hypothyroidism.

Additionally, there was a significant increase in plasma total TAC in hypothyroid rats treated with sesame oil.

### 4.6 Histopathological results

Light microscopic examination of both the control and sham control groups for liver and heart tissues showed no significant differences; therefore, they were discussed together.

**FIGURE 9:**
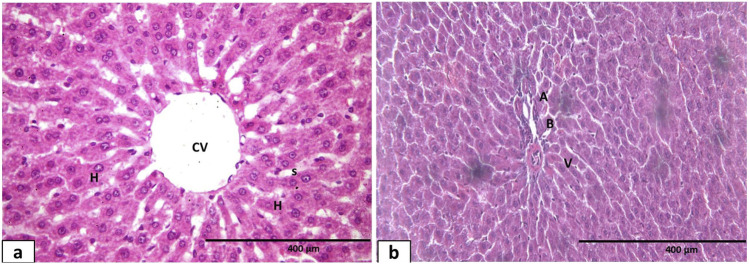
Photomicrograph of hematoxylin and eosin-stained sections of liver tissue of the control group **(a)** showing normal appearance of hepatic architecture. Hepatocytes (H) with vesicular nuclei and acidophilic cytoplasm arranged in anastomosing cords around a central vein (CV). Blood sinusoids are seen between the hepatocytes cords (s). Bi-nucleated hepatocytes could be seen (arrowhead). **(b)** Portal triad, hepatic artery (A), portal vein (V), and bile ductule (B).

**FIGURE 10:**
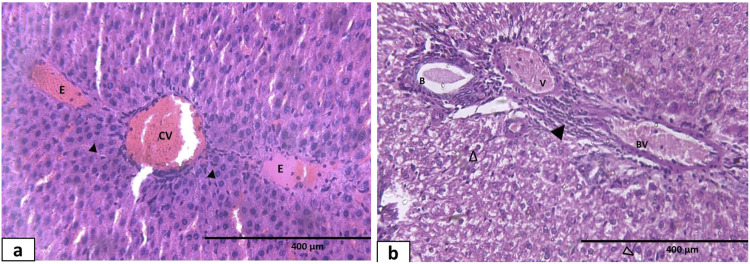
Photomicrograph of hematoxylin and eosin-stained sections of liver tissue of the hypothyroidism group **(a)** showing dilated central vein (CV), distorted hepatic architecture, and obliteration of sinusoids. Some hepatocytes appear with dark acidophilic cytoplasm and darkly stained nuclei (▲). Inflammatory exudates within the cytoplasm (E). **(b)** Dilatation and congestion of portal vein (PV) and dilatation of bile ductule (B) with periportal cellular infiltration (▲). Swelling and ballooning of some hepatocytes with vacuolated cytoplasm (Δ). Congested blood vessel was seen (X400).

Regarding the H&E sections taken from the right lobe of the liver of the control group, they showed a normal appearance of the hepatic architecture. The hepatocytes were arranged in anastomosing cords around a central vein, separated by blood sinusoids lined with flat endothelial cells. Hepatocytes appeared as polygonal cells with basophilic vesicular nuclei and acidophilic cytoplasm. A few cells appeared to have two nuclei. The margin of the liver lobule contained portal triad elements: a branch of the portal vein, a branch of the hepatic artery, and a bile ductule (Figures 9a, b). Conversely, the hypothyroid group displayed pronounced pathological changes, which were characterized by disorientation of the hepatocytes surrounding the congested central vein. Some of the hepatocytes near the central vein appeared swollen with darkly stained nuclei, and other cells seemed vacuolated. Inflammatory exudates were seen within the cytoplasm. Dilated, congested portal veins could be detected. In addition, periportal monocellular infiltration was markedly apparent (Figures 10a, b).

**FIGURE 11:**
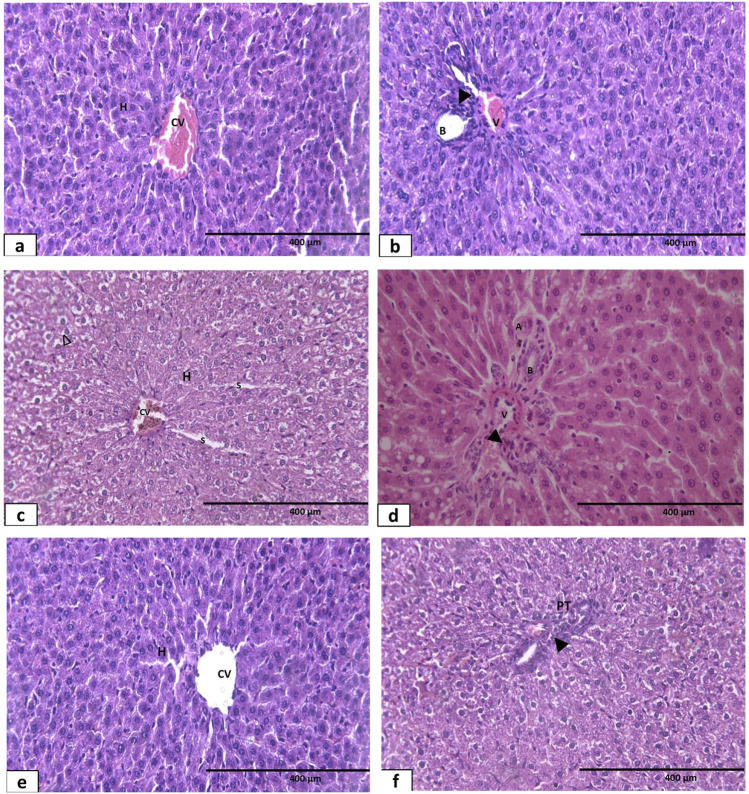
Photomicrograph of hematoxylin and eosin-stained sections of liver tissue of different groups: **(a, b)** sesame oil-treated group **(a)** showing that the hepatocyte cords were relatively arranged in an organized manner around the central vein (CV). Some hepatocytes have condensed nuclei and dark acidophilic cytoplasm (H). **(b)** Mild congested portal vein (V) and dilated bile duct (B) with some periportal cellular infiltration (▲). **(c, d)** L-thyroxine-treated group: **(c)** the liver architecture was partially restored, as some hepatocytes exhibited acidophilic cytoplasm (H), whereas others showed cytoplasmic vacuolations (Δ) with blood sinusoids in between. **(d)** Few inflammatory cell infiltrates (▲) around the portal tract; hepatic artery (A), portal vein (V), and bile ductule (B). **(e, f)** Combined treated group **(e)** showing almost normal appearance of the hepatocytes (H), and **(f)** minimal infiltration of inflammatory leucocytes (▲) is seen around the portal tract (PT) X400.

The liver sections of the sesame oil-treated group showed a mild improvement in certain lobules of the liver, where the hepatocyte cords were relatively arranged in an organized manner around the central veins. Many hepatocytes had pale acidophilic cytoplasm and spherical, vesicular nuclei (Figure 11a). Mild portal venous congestion with little periportal cellular infiltration was still noticeable (Figure 11b). In the thyroid-treated group, H&E-stained sections showed that the liver architecture was partially restored, as some hepatocytes exhibited acidophilic cytoplasm separated with blood sinusoids, whereas others showed cytoplasmic vacuolations (Figure 11c). Additionally, few inflammatory cell infiltrates and congested portal tract were still noted (Figure 11d).

Concomitant administration of thyroid and sesame oil showed noticeable improvement in the liver tissue’s structure. The hepatocytes had an almost normal appearance. Organized branching and anastomosing cords radiated from the central veins. Endothelium layers that were still intact lined the central veins (Figure 11e). The hepatic artery, portal vein, and bile ductule were visible in the portal regions, and there was no longer any congestion or mononuclear cellular infiltration (Figure 11f).

**FIGURE 12:**
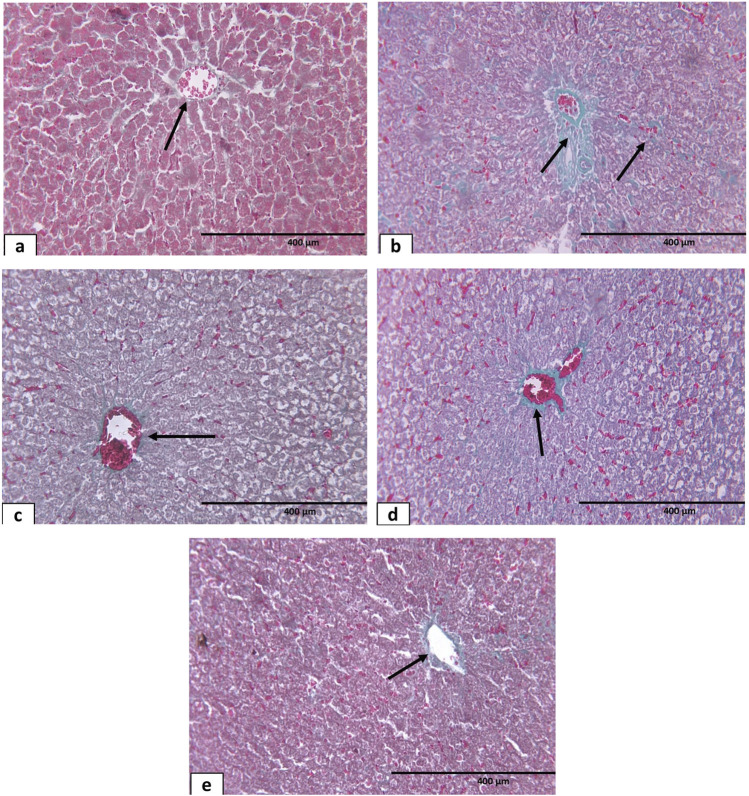
Photomicrograph of Masson’s trichrome-stained sections of liver tissue of different groups: **(a)** control group showing few collagen fibers (↑) around the central vein and in between the hepatic cords. **(b)** Hypothyroid group: marked increase in collagen fibers (↑) in between hepatocytes and around the central vein. **(c, d)** Sesame oil- and L-thyroxine treated groups, respectively, showing few collagen fibers (↑) around CV and in between the hepatic cords. **(e)** Combined treated group showing apparent normal collagen fibers around the central vein (↑) X400.

In Masson trichrome staining in the control and sham groups, there was deposition of a minimal amount of green-stained collagen fibers within the perisinusoidal spaces between the hepatic cords and around the central vein representing the normal hepatic fibrous stroma (Figure12a). Observation of Masson trichrome-stained liver sections in the hypothyroidism group showed an apparent increase in the deposition of green-stained collagen fibers around both the central veins and extending to the perisinusoidal spaces (Figure 12b). Both the sesame oil-treated group (Figure 12c) and L-thyroxine-treated group (Figure. 12d) showed apparent decrease in the deposition of collagen fibers around the central vein; however, it was more pronounced in the L-thyroxine-treated group. The combined treated group showed small amounts of deposition of collagen fibers around the central vein (Figure 12e).

**FIGURE 13:**
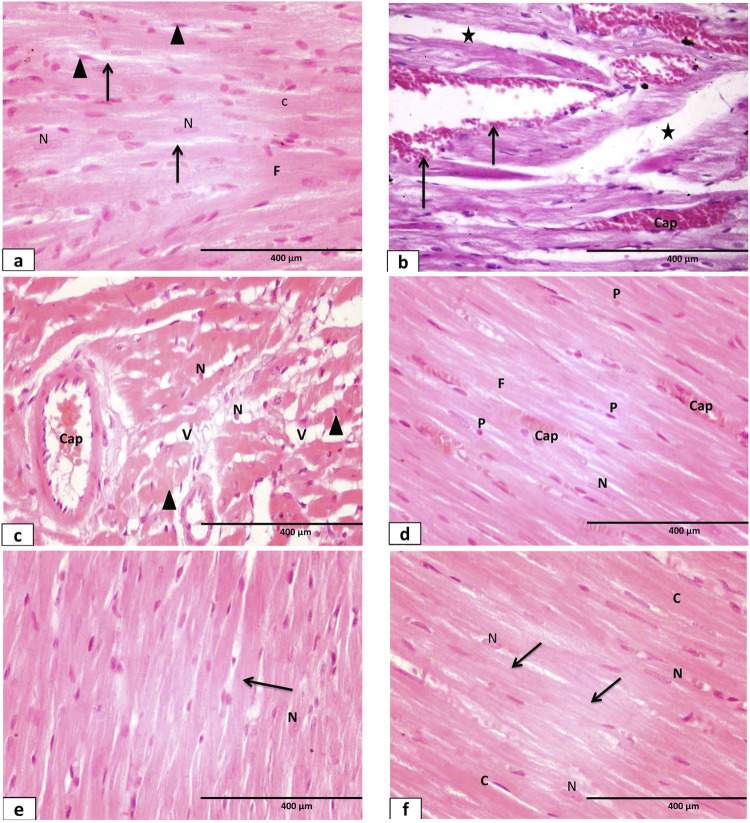
Photomicrographs of hematoxylin and eosin-stained longitudinal sections of the myocardium of the left ventricle of different groups: **(a)** control group showing normal structure of the myocardium formed of branching and anastomosing cardiac muscle fibers (F) with centrally located oval nuclei (N) and acidophilic cytoplasm (C). The cardiac muscle fibers are surrounded by delicate connective tissue endomysium (arrows) with dark oval nuclei of fibroblasts (arrow heads). **(b, c)** Hypothyroid group showing disturbed muscle structure with severe interstitial hemorrhage (arrows), engorged capillaries (Cap), and widened endomysium (star), along with fragmentation of muscle fibers (arrow head), darkly stained pyknotic nuclei (N), and vacuolations of the cytoplasm (V). **(d)** L-thyroxine-treated group showing apparently normal architecture of cardiac muscles with almost regular cardiomyocytes (arrow) and some central oval vesicular nuclei (N). **(e)** Sesame oil treated-group showing near-normal architecture of cardiac muscle fibers (F) with some central oval vesicular nuclei (N). Notice the engorgement of blood capillaries (Cap) and pyknosis of several nuclei (P). **(f)** Combined treated group showing the almost normal architecture of cardiac muscles with branching and anastomosing muscle fibers (arrow), central oval vesicular nuclei (N), and acidophilic cytoplasm (C) X400.

Sections obtained from the left ventricle of the control and sham control groups showed normal histological appearance of the myocardium, composed of branching and anastomosing cardiac muscle fibers with central oval vesicular nuclei and acidophilic cytoplasm. Cardiac muscle fibers were surrounded by delicate connective tissue endomysium with dark oval nuclei of fibroblasts (Figure 13a). On the other hand, the hypothyroid group revealed disturbed cardiac muscle structure in the form of massive interstitial hemorrhage, engorged capillaries, and widened endomysium. Darkly stained pyknotic nuclei, fragmented muscle fibers, and vacuolations of the cytoplasm were also detected (Figures 13b, c).

Sesame oil-treated group sections also showed near-normal architecture of cardiac muscle fibers, with some central oval vesicular nuclei. Engorgement of blood capillaries and pyknosis of many nuclei were detected (Figure 13d). Sections of the L-thyroxine-treated group showed normal architecture of cardiac muscles with almost regular cardiomyocytes and some central oval vesicular nuclei (Figure 13e).

Examination of the combined treated group sections revealed almost normal architecture of cardiac muscles with branching and anastomosing muscle fibers, central oval vesicular nuclei, and acidophilic cytoplasm (Figure 13f).

**FIGURE 14:**
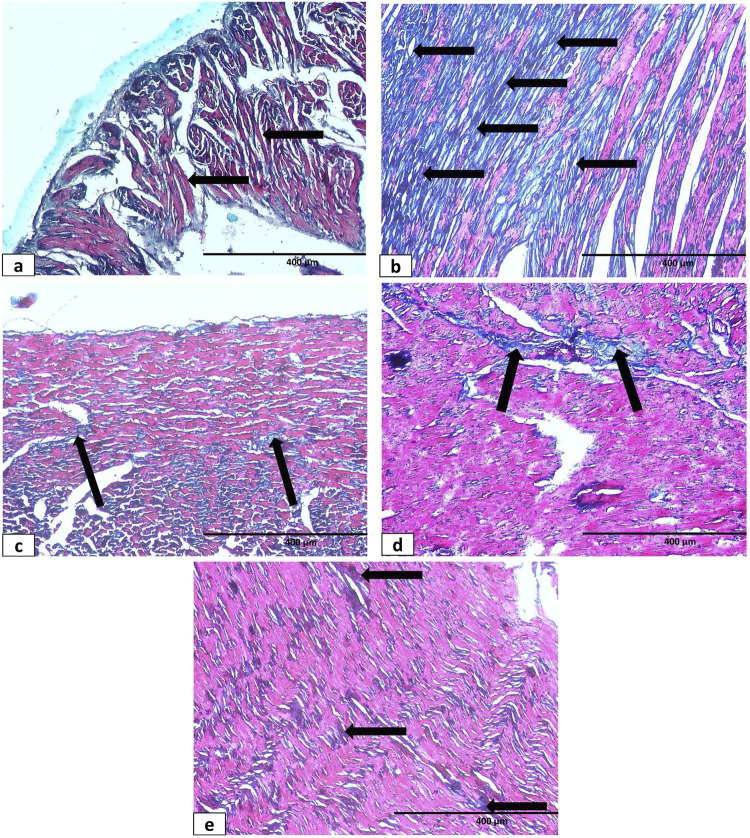
Photomicrographs of Masson’s trichrome-stained sections of cardiac muscles of different groups: **(a)** control group showing minimal endomysial collagen deposition (↗). **(b)** Hypothyroid group showing marked increase of collagen fibers (↗). **(c)** L-thyroxine-treated group showing apparent decrease in collagen deposition (↗). **(d)** Sesame oil-treated group showing apparent decrease in collagen deposition (↗). **(e)** Combined treated group showing minimal collagen deposition (↗) X400.

Masson trichrome-stained sections showed the amount of collagen deposition between cardiomyocytes in different groups; regarding the control and sham control groups, there was a negligible amount of collagen deposition (Figure 14a). In contrast, collagen deposition was massive in the hypothyroid group (Figure 14b). The sesame oil-treated group and L-thyroxine-treated group showed apparent decrease in collagen deposition compared to the hypothyroid group (Figures 14c, d). In addition, the combined treated group revealed a small amount of collagen deposition (Figure 14e).

#### 4.6.1 Immunohistochemical results

**FIGURE 15:**
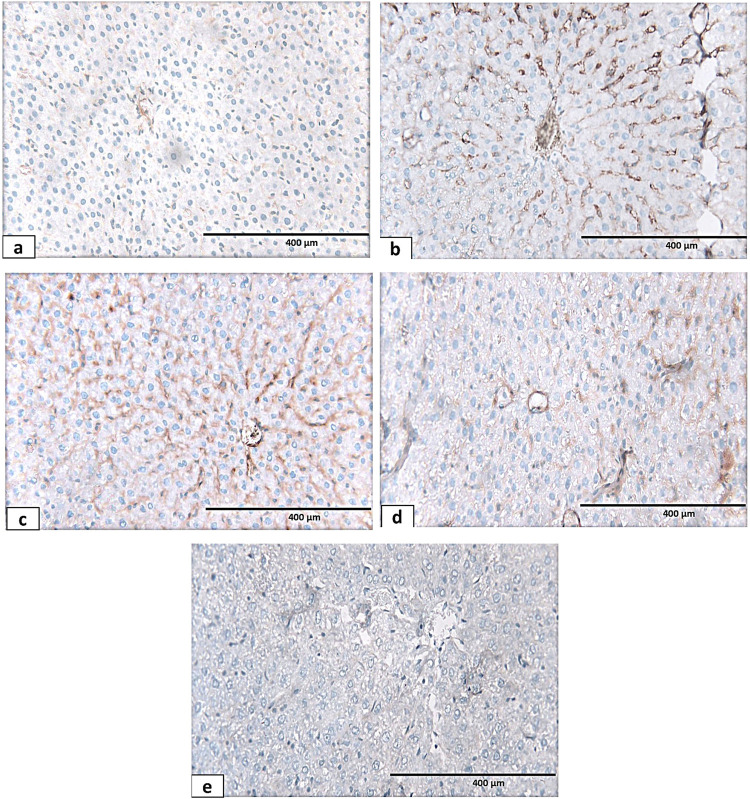
Photomicrograph of CD 68 immunohistochemical-stained sections of the liver tissue of different groups: **(a)** control group showing positive immunoreactivity of CD68 antibody in few Kupffer cells. **(b)** Hypothyroid group showing numerous CD68-positive Kupffer cells around the central vein and lining the hepatic sinusoids. **(c, d)** Sesame oil- and L-thyroxine-treated groups, respectively, showing the CD68-positive Kupffer cells. **(e)** Combined treated group showing few scattered positively stained Kupffer cells for CD68 X400.

To elucidate the functions of Kupffer cells in liver fibrosis, a specific Kupffer cell marker, CD68, was used to monitor Kupffer cell activation. The examination of CD68 immunostaining of liver sections showed few Kupffer cells that had positive immunoreaction for CD68 in the liver sections of the control and sham groups (Figure 15a). Many Kupffer cells with positive brown cytoplasmic reactions for CD68 in the hypothyroid group were detected (Figure 18b). In addition, positive immunoreaction for CD68 was detected similarly in many Kupffer cells in both sesame oil- (Figure 15c) and L-thyroxine-treated groups (Figure 15d). Meanwhile, in the combined treated group, few scattered positively stained Kupffer cells for CD68 were detected (Figure 15e).

**FIGURE 16:**
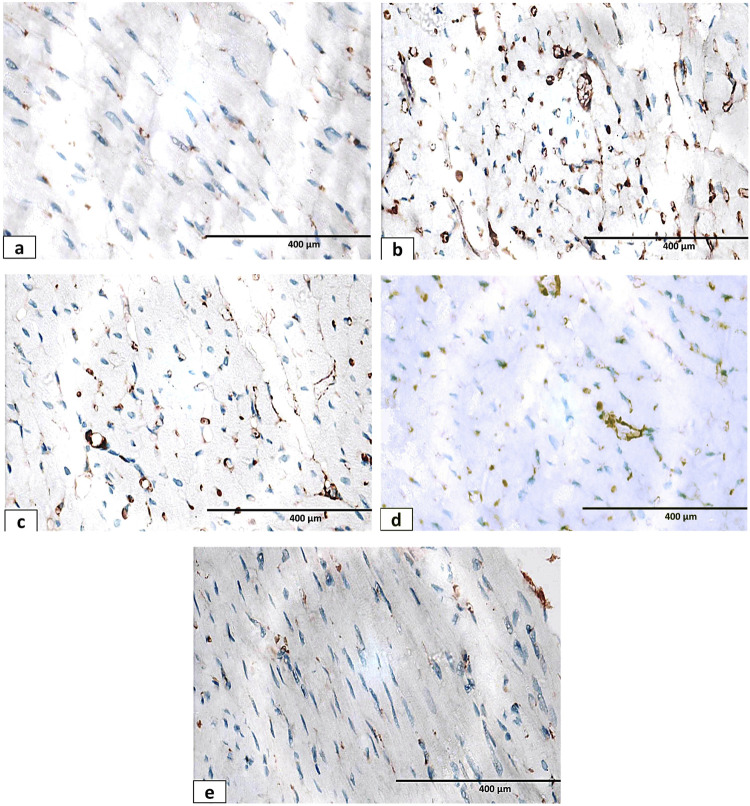
Photomicrographs of CD117 immunohistochemistry-stained sections of cardiac muscle of different groups: **(a)** control group: normal expression of CD117-positive mast cells. **(b)** Hypothyroid group showing an apparent massive increase in the expression of CD117-positive mast cells. **(c)** L-thyroxine-treated group showing an apparent decrease in the expression of CD117-positive mast cells. **(d)** Sesame oil-treated group showing an apparent decrease in the expression of CD117-positive mast cells. **(e)** Combined treated group showing an apparent near-normal expression of CD117-positive mast cells X400.

Immunohistochemical staining of CD117 was used for the identification of the stem/progenitor cells in the heart. CD117-positive cells appeared as dark brown dots. The distribution of CD117-positive cells showed an apparently marked increase in the hypothyroid group (Figure 16a) compared to the control group (Figure 16b). Regarding the L-sesame oil- (Figure 16c) and thyroxine-treated (Figure 16d) groups, there was a moderate distribution of CD117-positive cells. The mixed-treated group showed apparent minimal distribution of CD117-positive cells (Figure 16e).

#### 4.6.2 Morphometric results

**FIGURE 17:**
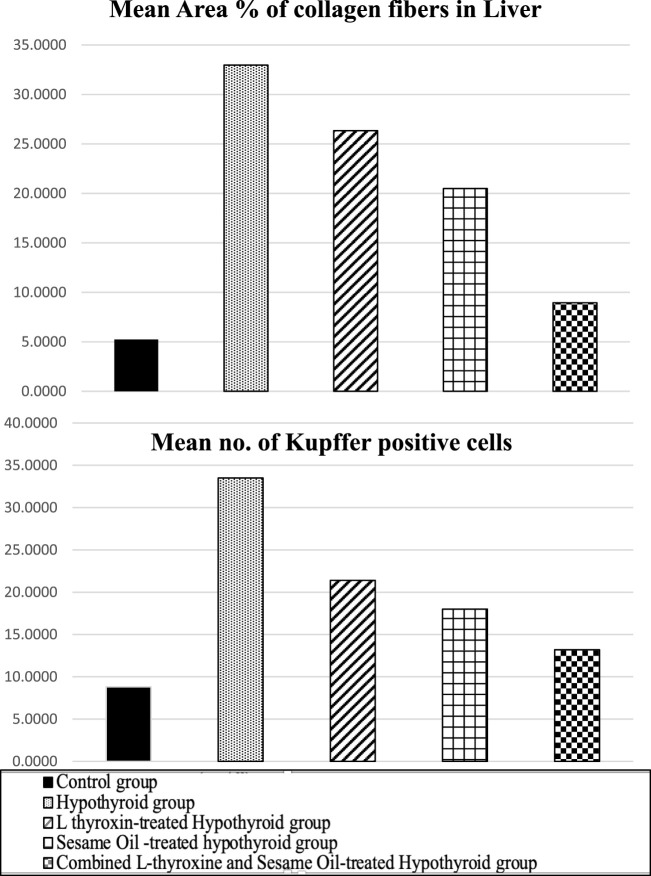
Mean area percentage of collagen fibers in the liver and the mean number of Kupffer-positive cells in the different studied groups.

Liver sections showed that the mean area % of collagen increased with high significance in the hypothyroidism groups compared to the control groups. A highly significant decrease was recorded in the sesame oil- and L-thyroxine-treated groups compared to the hypothyroidism group. There was no relatively significant difference between the sesame oil- and L-thyroxine-treated groups. In addition, a highly significant decrease was recorded in the combined treated group when compared to the hypothyroidism group, as observed in Table 2 and Figure 17.

**TABLE 2:** Comparison among the different experimental groups regarding the mean area % of collagen fibers and the mean number of Kupffer-positive cells in liver sections.

Groups	Control group	Hypothyroid group	Sesame oil-treated hypothyroid group	L-thyroxine-treated hypothyroid group	Combined treated hypothyroid group
Area % of collagen fibers	5.24 ± 1.88	32.97 ± 8.17^a^	26.34 ± 3.67^a^	20.49 ± 3.47^a^ ^,^ ^b^	8.95 ± 1.64^b^
Mean no. of Kupffer-positive cells	8.8 ± 3.61	33.5 ± 5.73^a^	21.4 ± 3.02^a^	18 ± 3.26^a^ ^,^ ^b^	13.20 ± 3.01^b^

Results are expressed as the mean ± SD.

^a^ P < 0.001 vs. the control group (highly significant difference) in one-way ANOVA, followed by post hoc Bonferroni test.

^b^ P < 0.001 vs. the hypothyroidism group (highly significant difference) in one-way ANOVA, followed by post hoc Bonferroni test.

The mean number of CD68-positive Kupffer cells in CD68-stained sections exhibited a highly significant increase in the hypothyroid group compared to the control group. The sesame oil- and L-thyroxine-treated groups showed a significant decrease in the mean number of CD68-positive Kupffer cells as compared to the hypothyroid group. A highly significant decrease in the mean number of CD68-positive cells was recorded in the combined treated group as compared to the hypothyroid group, as observed in Table 2 and Figure 17.

**FIGURE 18:**
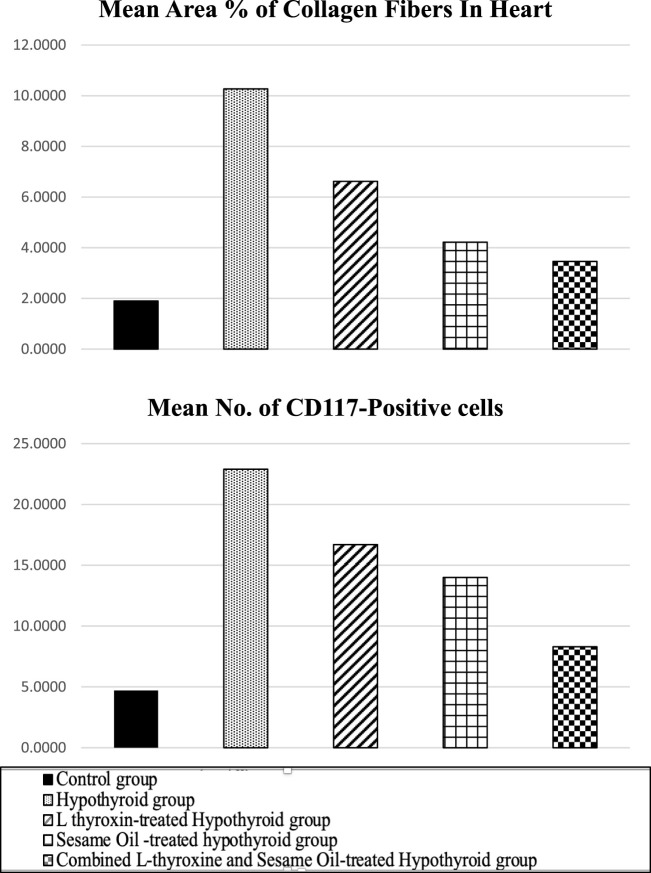
Mean area percentage of collagen fibers in the heart and the mean number of CD117-positive cells in heart in the different studied groups.

Regarding sections of the left ventricle of the heart, the mean area % of endomysial collagen deposition showed a highly significant increase in the hypothyroid groups compared to the control. There was no relatively significant difference between the sesame oil- and L-thyroxine-treated groups. The combined treated group exhibited a highly significant increase compared to the hypothyroidism group, as demonstrated in Table 3 and Figure 18.

**TABLE 3:** Comparison among the different experimental groups regarding the mean area % of collagen fibers and the mean number of CD117-positive progenitor cells in heart sections.

Groups	Control group	Hypothyroid group	Sesame oil-treated hypothyroid group	L-thyroxine-treated hypothyroid group	Combined treated hypothyroid group
Area % of collagen fibers	19000 ± 92376	10.27± 1.79^a^	6.62± 2.13271^a^	4.22± 1.03258^a^ ^,^ ^b^	3.46± 8.13 ^b^
Mean no. of CD117-positive progenitor cells	4.7± 1.25	22.9± 4.58^a^	16.7± 2.83^a^	14± 2.75^a^ ^,^ ^b^	8.3± 2.21 ^b^

Results are expressed as the mean ± SD.

^a^ P < 0.001 vs. the control group (highly significant difference) in one-way ANOVA, followed by post hoc Bonferroni test.

^b^ P < 0.001 vs. the hypothyroidism group (highly significant difference) in one-way ANOVA, followed by post hoc Bonferroni test.

The mean number of CD117-positive progenitor cells of the left ventricle-stained section showed highly significant increase in the hypothyroid group compared to the control group. The sesame oil- and L-thyroxine-treated groups showed a significant decrease in the mean number of CD117-positive cells compared to the hypothyroid group. The combined treated group, exhibited a highly significant decrease in the mean number of CD117-positive cells compared to the hypothyroid group, as shown in Table 3 and Figure 18.

## 5 Discussion

PTU administration, herein, caused primary hypothyroidism, in accordance with Bhanja and Jena (2013), through inhibiting thyroid hormone synthesis (Parmar and Kar, 2009) and preventing the peripheral de-iodination of T4 to T3 (Yi et al., 1997). Meanwhile, L-thyroxine treatment, herein, restored the normal thyroid status, similar to the results of Shahrivar et al. (2016). In addition, sesame oil treatment in hypothyroid rats restored the euthyroid status, which is a novel finding in this study. It could be explained, according to Sharma et al. (2014), as probably by being a source of tyrosine, an essential factor for thyroid hormone synthesis, and by containing healthy fatty acids that maintain the normal function of the thyroid gland.

The persistently higher TSH levels in all the treated hypothyroid groups in this study, despite regaining euthyroid status, agree with Nicoloff and Spencer (1992). These findings could be due to the loss of the usual reverse relationship between the serum levels of TSH and T4 in hypothyroid cases (Refetoff et al., 1993).

In this study, we demonstrated that a combination of sesame oil and L-thyroxine treatment restored the euthyroid status and improved the effects of hypothyroid-induced altered cardiovascular and liver functions for the first time, to the best of our knowledge.

The hypothyroid group, herein, had dyslipidemia, in agreement with the findings of Murgod and Soans (2012), which has been attributed to the ability of thyroid hormones to increase the synthesis, mobilization, and storage of triglycerides in adipose tissue, as well as the lipoprotein lipase activity (Pucci et al., 2000). The observed hypothyroidism-induced upregulated SCD1 gene expression in liver tissues agrees with the finding of Hashimoto et al. (2013). SCD1, a lipogenic and rate-limiting enzyme in monounsaturated fatty acid synthesis, regulates hepatic lipogenesis and lipid oxidation (Dobrzyn and Ntambi, 2005). Thus, it could be one of the underlying mechanisms of hypothyroidism-induced dyslipidemia. In addition, the observed lowered hepatic LDL-R concentration in hypothyroid individuals could explain the coexistent hypercholesterolemia, in accordance with the findings of Jiskra et al. (2007). The hypercholesterolemia in hypothyroidism was attributed to the lowered LDL-receptor’s (LDL-R) concentration reducing the catabolism of LDL-cholesterol (Abbas et al., 2008).

Dyslipidemia was improved in L-thyroxine-treated hypothyroid rats in the current study despite an insignificant rise in hepatic LDL-R concentrations. This could be explained by the presence of a non-LDL-R pathway, resulting in lowering liver ApoB production by thyroid hormones, according to Goldberg et al. (2012). In addition, thyroid receptor-β, which is present in hepatic tissues, could affect LDL-R transcription, thereby elevating cholesterol uptake and cholesterol synthesis (Grover et al., 2005).

The lipolytic effects of sesame oil, similar to the findings of Alipoor et al. (2012), could be attributed to its contents of oleic acid and linoleic acid, which reduce the absorption of lymphatic cholesterol (Satchithanandam et al., 1996), and to sesamin, which lowers hepatic lipogenesis (Ide et al., 2013).

In addition, the higher hepatic LDL-R concentrations induced by sesame oil, which was demonstrated for the first time, could partially explain the amelioration of dyslipidemia by sesame oil treatment. Interestingly, the downregulated hepatic SCD1 gene expression by sesame oil treatment could be another mechanism of its lipid-lowering effects in the hypothyroid status. In support of this suggestion, Pen˜alvo et al. (2006) found that sesamin had hypolipidemic effects in LDL-R-deficient female mice.

On the other hand, combined treatment with L-thyroxine and sesame oil, herein, improved dyslipidemia, which may be through their synergistic effects on LDL receptors and SCD1 downregulation. This synergism was more prominent in lowering plasma LDL-cholesterol together with the suppression of hepatic SCD1 gene expression and the simultaneous increase of hepatic LDL-R concentrations.

Regarding the cardiovascular changes, the hypothyroid group had systolic hypotension and diastolic hypertension, whereas the administration of either L-thyroxine alone or sesame oil alone limited the systolic hypotension despite persistent higher diastolic blood pressure.

Blood pressure changes in hypothyroid rats were previously explained by lowered cardiac output due to impaired relaxation of vascular smooth muscle and the reduced availability of endothelial nitric oxide. The subsequent higher arterial stiffness enhances systemic vascular resistance. In addition, the cardiac structure was found to be altered by thyroid hormone deficiency in the form of lowered expression of sarcoplasmic reticulum Ca^2+^-ATPase and higher expression of phospholamban, which inhibited ATPase in the study of Klein and Danzi (2007). Thyroid hormone was also found to affect the renin–angiotensin–aldosterone system through facilitating the synthesis of angiotensinogen in the liver. Thus, diastolic blood pressure is elevated, pulse pressure is reduced, and renin levels are reduced in hypothyroidism (Klein and Danzi, 2007).

The restored normal systolic blood pressure values after L-thyroxine treatment in the current study are in accordance with those in the study by Pingitore et al. (2012), as T3 enhances inotropy and chronotropy. Furthermore, sesame oil treatment restored normal systolic blood pressure, which agrees with the study by Liu et al. (2014). Although either L-thyroxine or sesame oil had little effect in improving the diastolic blood pressure, this could be attributed to the persistent slightly elevated plasma levels.

Meanwhile, hypothyroidism-induced cardiac changes could be caused by the present oxidative stress (Gupta et al., 2002) and/or the associated inflammatory process (Syamsunder et al., 2017). Thus, the improvement in cardiac function observed in this study following sesame oil treatment could be mediated by counteracting oxidative stress, as reported by Liu and Liu (2017), as well as affecting the renin–angiotensin system; however, this was not directly assessed in this study. Moreover, another possible mechanism of such improving effects of sesame oil could be through being a source of vitamin E, which enhances enzymatic and nonenzymatic antioxidants (Chandrasekaran et al., 2014). Furthermore, the antioxidant effects of sesame oil may be due to its content of sesamin and sesamolin, phytosterols, and flavonoids (Reshma et al., 2010), in addition to its anti-inflammatory effects (Wan et al., 2015).

Interestingly, combined L-thyroxine and sesame oil-supplemented hypothyroid rats, herein, had almost normal diastolic blood pressure values, which reflect the better blood pressure control induced by the simultaneous supplementation of sesame oil and the deficient hormone in this group. This could be due to their synergistic lipid-lowering effects on dyslipidemia, probably by increasing hepatic LDL-R concentrations, and to their restorative effects in thyroid hormone synthesis.

The hypothyroidism-induced electrocardiographic changes observed in this study agree with those reported by Stephan et al. (2003) and Kweon et al. (2007). Such ECG changes could be explained by the prolonged duration of the ventricular action potential due to the lowered activity of voltage-gated potassium channels (Nathaniel et al., 1994). The QT dispersion, caused by varied myocardial repolarization, was frequent in hypothyroidism, resulting in ventricular arrhythmias (Kweon et al., 2007). In addition, the systolic hypotension and bradycardia present in the hypothyroid group could be attributed to autonomic neuropathy in the form of a higher vagal tone, according to Xing et al. (2001).

In addition, it was suggested that such ECG changes in the hypothyroid group could be related to left ventricular diastolic dysfunction (Dillmann, 2010) and the coexistent dyslipidemia (Mansourian, 2012). On the contrary, Fadeyev et al. (2006) did not find any differences in the blood lipids, left ventricular diastolic function, and heart rate in the hypothyroid state. Meanwhile, L-thyroxine treatment, herein, improved such ECG abnormalities, which agrees with those reported by Stephan et al. (2003) and Bakiner et al. (2008). This could be explained by L-thyroxine’s ability to restore normal sympathetic tone to the heart in addition to its lipolytic effects.

On the other hand, sesame oil affected such ECG abnormalities and blood pressure changes, probably by alleviating hypokalemia, despite not being assessed in this study. As previously reported in literature, a prolonged QT interval was associated with potassium insufficiency (Akita et al., 1998), causing hypertension (Asmar et al., 2012).

In addition, hypothyroidism, in this study, caused higher plasma levels of cardiac enzymes, which is in agreement with Doran and Wilkinson (1975), suggesting myocardial damage. Such higher cardiac enzymes in hypothyroid rats could be due to the coexistent dyslipidemia, according to Cohen et al. (1996). Therefore, all the treated hypothyroid groups had higher cardiac enzymes in the plasma, denoting the limitation of myocardial damage, probably through alleviating the associated dyslipidemia, inflammation, and oxidative stress.

The altered cardiac morphology observed in hypothyroid rats supports the higher cardiac enzymes in these rats. In the current study, there was a disturbed histopathological structure of the cardiac muscle in all the hypothyroid groups. These results may arise from microvascular impairment caused by low thyroid function (Sara et al., 2015). Moreover, there was a massive interstitial hemorrhage and engorged capillaries, which could be due to inflammation. This may cause vascular leakage, widened endomysium, and fragmentation of muscle fibers that may be related to edema and accumulation of mucopolysaccharides resulting from necrotic changes (Farag et al., 2024). Additionally, multiple pyknotic nuclei and vacuolated cytoplasm were detected in the present work, which could be attributed to oxidative stress and apoptosis that cause pyknosis (Abdel-Daim et al., 2017) and lipid accumulation, swelling of cellular mitochondria, and dilatation of sarcoplasmic reticulum, resulting in cytoplasmic vacuolation (Lind et al., 2021).

The previous findings were supported by Masson’s trichrome-stained sections, which showed a highly significant increase in the area percentage of collagen fibers. This increase may be due to the proliferation, migration, and trans-differentiation of fibroblasts into myofibroblasts, which produce significant amounts of interstitial collagen, all induced by myocardial injury (Abo Elnasr et al., 2024). Similarly, there was a significant increase in the number of CD117-positive cardiac progenitor cells. In support, Di Meglio et al. (2010) demonstrated that the number of CD117-positive cells increased in the pathological hearts; these primitive cardiac CD117-positive cells are known to give rise to cardiomyocytes.

Higher plasma CK-MB levels contrast with the findings of Minutiello (1993), who found that its levels were not associated with acute myocardial infarction in hypothyroidism. Buschmann et al. (2007) found that the troponin I level was elevated without any myocardial damage in a hypothyroid patient. However, cardiac fiber disruption was noted in hypothyroid rats in our study, which could be evidence of cardiac damage and higher cardiac enzymes in the plasma. In support of this suggestion, the higher plasma AST and CK-MB levels in the hypothyroid group disagree with the findings of Bobadilla et al. (2001).

In this study, oxidative stress was prominent in the hypothyroid group, similar to that reported by Petrulea et al. (2010), although this finding disagrees with that of Venditti et al., (1997). However, Coria et al. (2009) found no change in lipid peroxidation in the hypothyroid status. This discrepancy could be attributed to the duration of the hypothyroid status and the use of different experimental designs, or varied animal species (Shahrivar et al., 2016).

On the other hand, the higher plasma IL-6 level in the hypothyroid group, herein, reflects the inflammatory process in such a state, which is in line with the findings of Hajje et al. (2014). L-thyroxine treatment attenuated the oxidative stress and the inflammation, to a lesser extent, in the hypothyroid rats, which is similar to the findings of Hicks et al. (1992). The link between oxidative stress and hypothyroidism was described as a vicious circle as hypothyroidism could aggravate the oxidative stress status, probably through the production of interleukins, which might lower deiodinases expression, causing further oxidative stress. Thus, thyroid hormones modulated the antioxidant levels and inflammatory mediators (Mancini et al., 2016), which were observed in this study.

Sesame oil administration induced improvement of dyslipidemia and cardiac dysfunction, herein, through its antioxidant and anti-inflammatory properties, which agree with Periasamy et al. (2014). In line with Aluganti et al. (2015), it is suggested that a sesame oil-enriched diet could be an effective non-pharmacological treatment for atherosclerosis by controlling inflammation, reducing plasma IL-6 levels, and regulating lipid metabolism.

Furthermore, hepatic lobular damage and elevated plasma liver enzymes in the hypothyroid group, herein, confirmed liver damage, denoting oxidative stress. Similarly, Mullur et al. (2014) demonstrated that hypothyroidism induced oxidative stress in the mouse liver, which is most likely the consequence of the effects of thyroid hormones on cellular metabolism. The hypothyroid group had disoriented hepatocytes surrounding a congested central vein, some swollen hepatocytes with darkly stained nuclei, and other cells that seemed vacuolated, in addition to inflammatory exudates and mononuclear cellular infiltration. These alterations suggested the development of an inflammatory degenerative process in the liver. Demir et al. (2016) suggested that hepatic degeneration can be caused by a lack of thyroid hormones. Therefore, hepatic steatosis may exist in hypothyroidism, even in subclinical cases, which is related to MALD in a dose-dependent manner (Tanase et al., 2020). Furthermore, Ayuob et al. (2019) suggested that hypothyroidism is independently linked to MALD regardless of the common metabolic risk factors.

Moreover, fibrosis was evident in the hypothyroid group, which manifested as an increase in the deposition of collagen fibers around both the central veins and extending to perisinusoidal spaces. Thyroid dysfunction can promote fibrosis through pathways involving altered collagen gene expression, collagen deposition, and fibrogenic cytokine activity (Rastegar-Moghaddam et al., 2023). Similarly, it was shown that hypothyroidism can exacerbate liver fibrosis, which can be reversed by thyroid hormone therapy (Rahadini and Rahadina, 2022).

All experimental groups, including the hypothyroid group, revealed the presence of aggregations of mononuclear inflammatory cells in the hepatic parenchyma composed mainly of Kupffer cells, as detected by CD68 immunostaining. These aggregations were suggested to be a type of delayed type IV hypersensitivity granulomatous reaction involving perivascular accumulation of T-helper lymphocytes and Kupffer cells (Fattin et al., 2017). Furthermore, Kupffer cells activate hepatic stellate cells by releasing mediators such as pro-inflammatory cytokines and ROS, which are critical for liver inflammation. Hepatic stellate cells, in turn, promote liver fibrosis by releasing connective tissue growth factor and sequestering platelet-derived growth factor receptor-α (Kostallari et al., 2018).

Therefore, impaired liver function was observed in hypothyroid rats in this study, as indicated by elevated plasma ALT and AST activities and hepatic lobular damage, all of which improved in the treated hypothyroid groups. The higher circulating liver enzymes may be due to higher extracellular leakage from injured hepatocytes, probably by the coexistent dyslipidemia (Christ-Crain et al., 2004). The findings of higher liver enzyme activities, hepatic steatosis, and inflammation could also be explained by the effects of thyroid hormones on the intrahepatic lipid metabolism, such as fatty acid β-oxidation and fatty acid delivery to mitochondria (Sinha et al., 2012). Similarly, Ittermann et al. (2012) reported that nonalcoholic fatty liver disease was present in cases of overt hypothyroidism.

The improvement of hepatic steatosis and dyslipidemia by L-thyroxine administration follows the study by Gardner et al. (2011). In line with this, Cable et al. (2009) found that hepatic selective thyroid receptor-β agonist lowered hepatic steatosis and circulating levels of FFA and TG. In addition, dyslipidemia in the hypothyroid group could be related to hydropic degeneration and steatosis in hepatic tissues, as mentioned by Parmar and Kar (2008). However, Cano-Europa et al. (2010) reported that methimazole could induce hepatic cell damage, whereas surgically induced hypothyroidism did not cause such hepatic injury. This discrepancy could be attributed to the different rat strains and/or the different experimental procedures.

The impaired liver functions and disrupted hepatic structure in the hypothyroid group could also be attributed to coexistent oxidative stress and inflammation. On a similar note, Yilmaz et al. (2003) found that hypothyroidism caused oxidative stress in the liver and heart, causing an imbalance between ROS and endogenous antioxidant body defenses, with subsequent damage to cellular macromolecules (Gupta et al., 2002).

L-thyroxine administration moderated such oxidative stress in hypothyroid rats through normalizing the plasma MDA level and plasma TAC, in agreement with Varghese et al. (2001). However, Chattopadhyay et al. (2010) demonstrated that oxidative stress in the hypothyroid status was not reversed with T3 treatment. This conflict may be attributed to the variable periods of hypothyroidism and thyroid hormone treatment, or due to different doses of thyroid hormone.

Hypothyroid treatment with sesame oil alone reduced ALT activity compared to the hypothyroid group despite still being higher than the control values; meanwhile, its combination with L-thyroxine normalized plasma ALT activity. These findings align with those of Lv et al. (2015), who suggested that the hepatoprotective effects of sesamin may result from its ability to reduce oxidative stress. Thus, when sesame oil was combined with thyroid hormone replacement, its hepatoprotective efficiency was enhanced.

The improvement in hepatic steatosis observed in the sesame oil-treated group in this study is consistent with findings by Periasamy et al. (2014). In line with the findings, sesame seeds affected hepatic fatty acid oxidation and serum triacylglycerol levels due to their antioxidant effects (Ide et al., 2015). Likewise, sesamol attenuated dyslipidemia, IL-6 levels, hepatic transaminases, and alkaline phosphatase, and it normalized the arterial pressure in a dose-dependent manner (Sharma et al., 2012). On the contrary, oral intake of sesame oil had no therapeutic effects on the raised liver enzymes and sinusoidal obstruction in monocrotaline-treated rats (Periasamy et al., 2013). This discrepancy may be due to the difference in sesame oil doses and/or the duration of its supplementation.

The ameliorated oxidative stress and improved liver functions and structure in sesame oil-treated hypothyroid rats, as observed in this study, are consistent with those of Soliman et al. (2015). These hepatoprotective effects of sesame oil could be attributed to the presence of the natural antioxidants sesamol, sesamolin, and gamma tocopherol (Corso et al., 2010).

Hypothyroidism leads to increased triglyceride levels and decreased HDL levels.

Sesame oil supplementation appears to moderate hepatic and lipid profile changes, reducing triglycerides and increasing HDL levels compared to rats without supplementation. Overall, hypothyroid rats exhibit a dyslipidemic profile (high TG and low HDL) that sesame oil partially improves, as shown by the estimated marginal means. In addition, sesame oil treatment reduces plasma AST and creatine kinase levels, which are elevated due to hypothyroidism. This suggests a protective normalizing effect of sesame oil against markers of liver damage that are commonly seen in hypothyroid rats. Crucially, there is a significant interaction effect between the thyroid status and sesame oil treatment, indicating that the effect of sesame oil differs based on the thyroid condition. Specifically, sesame oil significantly reduces the elevated AST and creatine kinase levels in hypothyroid rats, effectively normalizing these markers compared to untreated hypothyroid controls.

Thus, sesame oil treatment statistically alleviates biochemical disruptions caused by hypothyroidism, as confirmed by the interaction effect in the two-way ANOVA. This suggests that sesame oil modifies or counters hypothyroidism-induced changes rather than exerting uniform effects across thyroid statuses.

The higher TSH levels in the three treated hypothyroid groups may reflect not reaching an adequate replacement therapy or may require longer experimental periods to regain complete hypothalamus–pituitary–thyroid axis recovery. In line, The European Thyroid Association guideline recommended repeated TSH measurements separated by 2- to 3-month intervals in human subjects (Pearce et al., 2013); however, it is not performed in this study due to the collection of blood samples after sacrifice.

In addition, the elevated TSH levels may point to different levels of thyroid hormones *in vivo* in experimental rats during the restoration of normal thyroid control. On a similar note, Escobar-Morreale et al. (1996) suggested that it is hard to create normal levels of T3 in all tissues with a dose of L-thyroxine alone.

In conclusion, both L-thyroxine and sesame oil provided cardioprotective and hepatoprotective effects primarily by ameliorating oxidative stress. Sesame oil exhibited stronger lipolytic activity by enhancing hepatic LDL-receptor gene upregulation, and it also provided notable anti-inflammatory, antifibrotic, and antisteatotic effects in hypothyroid rats. Combining L-thyroxine with sesame oil yielded synergistic lipid-lowering effects and better diastolic blood pressure control. Therefore, sesame oil may offer additive therapeutic values in managing cardiovascular and hepatic complications associated with hypothyroidism in rats. Further studies on sesame oil in hypothyroidism are recommended to determine whether a sesame oil-enriched diet could reduce the required dose of L-thyroxine replacement therapy.

## 6 Limitation and recommendation

Although all the groups were provided with the same standard diet, food intake was not measured separately, which may have influenced the metabolic outcomes. This has now been highlighted as a limitation and a recommendation for future studies. It could be recommended that more mechanistic investigations are needed for further studies, specifically on the molecular levels.

## Data Availability

All data are available upon reasonable request from the corresponding author.
